# Persistent CSF Rhinorrhoea, Pneumocephalus, and Recurrent Meningitis Following Misdiagnosis of Olfactory Neuroblastoma

**DOI:** 10.1155/2010/312081

**Published:** 2010-08-05

**Authors:** Neil Barua, David A. Hilton, William Mukonoweshuro, Hisham Khalil, Louis Pobereskin

**Affiliations:** ^1^Department of Neurosurgery, Derriford Hospital, Plymouth PL6 5DH, UK; ^2^Department of Neuropathology, Derriford Hospital, Plymouth PL6 5DH, UK; ^3^Department of Neuroradiology, Derriford Hospital, Plymouth PL6 5DH, UK; ^4^Department of Otorhinolaryngology, Derriford Hospital, Plymouth PL6 5DH, UK

## Abstract

A 41-year-old female patient was admitted with streptococcal meningitis on a background of 5-month history of CSF rhinorrhoea. Imaging revealed an extensive skull base lesion involving the sphenoid and ethmoid sinuses, the pituitary fossa with suprasellar extension and bony destruction. Histological examination of an endonasal transethmoidal biopsy suggested a diagnosis of olfactory neuroblastoma. A profuse CSF leak occurred and the patient developed coliform meningitis. A second endonasal endoscopic biopsy was undertaken which demonstrated the tumour to be a prolactinoma. Following endonasal repair of the CSF leak and lumbar drainage, she developed profound pneumocephalus. The patient underwent three further unsuccessful CSF leak repairs. Definitive control of the CSF leak was finally achieved through a transcranial approach with prolonged lumbar drainage. This case illustrates some of the potentially devastating complications which can occur as a consequence of complex skull base lesions. A multidisciplinary approach may be required to successfully manage such cases.

## 1. Introduction

Traditionally, complex paranasal and anterior skull base lesions have been managed with either transcranial or transphenoidal microsurgical approaches. The application of endoscopy to neurological surgery and advances in neuronavigation have provided further options to skull base surgeons. One of the commonest complications of skull base lesions and their management is the occurrence of CSF leakage, which can result in the potentially fatal consequences of pneumocephalus and meningitis. Despite advances in microsurgical and endoscopic technology and expertise, definitive management of complex lesions and their resultant complications may require multidisciplinary team management and open transcranial neurosurgery. 

## 2. Case Report

**Figure 1:**
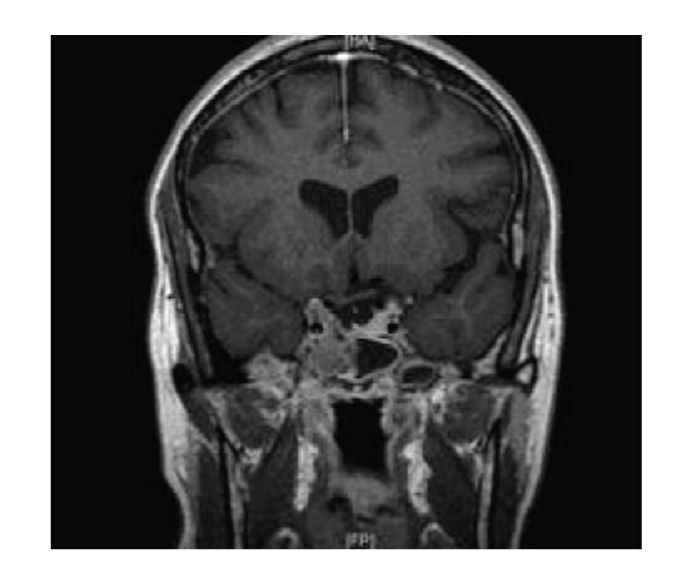
Postgadolinium coronal T1 weighted image showing an enhancing, soft tissue mass involving the right side of the sphenoid sinus and extending into the right cavernous sinus and into the suprasellar cistern.

**Figure 2:**
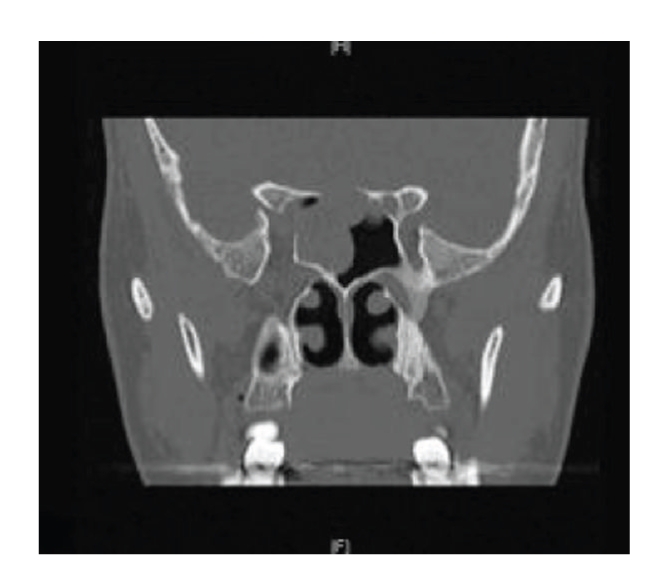
Coronal CT scan showing soft tissue mass in the sphenoid sinus with destruction of the roof of the sphenoid sinus.

A 41-year-old female patient was referred to the ENT outpatient clinic with 5-month history of clear fluid discharging from the nose. Fluid samples were sent for tau protein analysis and outpatient imaging was requested. Prior to completion of skull base imaging, the patient was admitted in an acute confusional state, with a nonblanching rash, pyrexia, and signs of meningism. A diagnosis of streptococcal meningitis was made following lumbar puncture and she was commenced on appropriate antibiotics. Magnetic resonance imaging demonstrated an extensive skull base lesion involving the sphenoid and ethmoid sinuses, pituitary fossa, and suprasellar region (Figure 1). Computed tomography of the sinuses revealed bony destruction (Figure 2).

An endonasal transethmoidal biopsy was undertaken, following which there was profuse CSF rhinorrhoea. Subsequently, the patient developed clinical signs of meningism with pyrexia, neck stiffness, photophobia, and deterioration in neurological status. CSF analysis revealed gram-negative coliforms and antibiotic treatment was commenced.

### 2.1. Transphenoidal and Endoscopic Endonasal Repair of CSF Leak

The patient was transferred to the regional neuroscience centre for joint neurosurgical and ENT management. On recovery from the second episode of meningitis, she underwent an endoscopic endonasal biopsy and repair of the anterior pituitary fossa and planum sphenoidale using layered fat graft and artificial dural substitute sealed with Tisseel fibrin sealant (Baxter Healthcare, UK). Histological analysis of this biopsy specimen confirmed the tumour to be a prolactinoma. Although her initial serum prolactin level was only 451 miu/mL (normal range 102–496), it did rise to 953 miu/mL in the immediate postbiopsy period. Following endocrine review, she was commenced on cabergoline.

**Figure 3:**
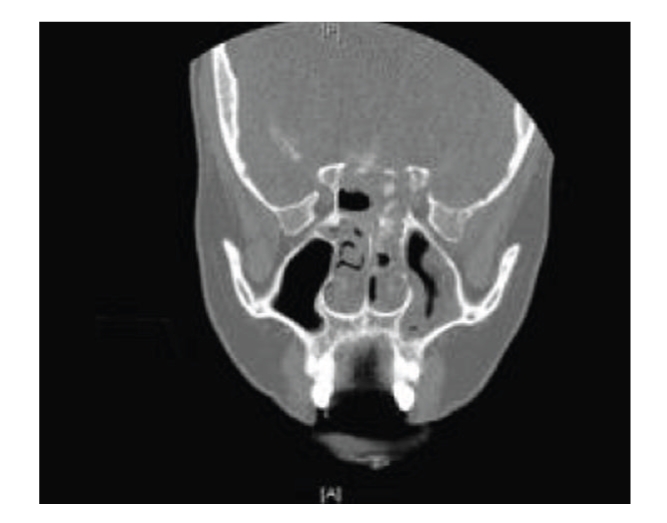
CT cisternogram showing a right-sided CSF leak through the roof of the sphenoid sinus.

Despite satisfactory intraoperative appearances, CSF rhinorrhoea recurred. A CT cisternogram was undertaken to further characterise the site of CSF leakage (Figure 3). Through a sublabial transphenoidal microsurgical approach, two further repairs of bony defects in the floor of the pituitary fossa and roof of the sphenoid sinus were undertaken using fascia lata and fat grafts sealed with Tisseel glue, along with a period of postoperative lumbar CSF drainage. 

**Figure 4:**
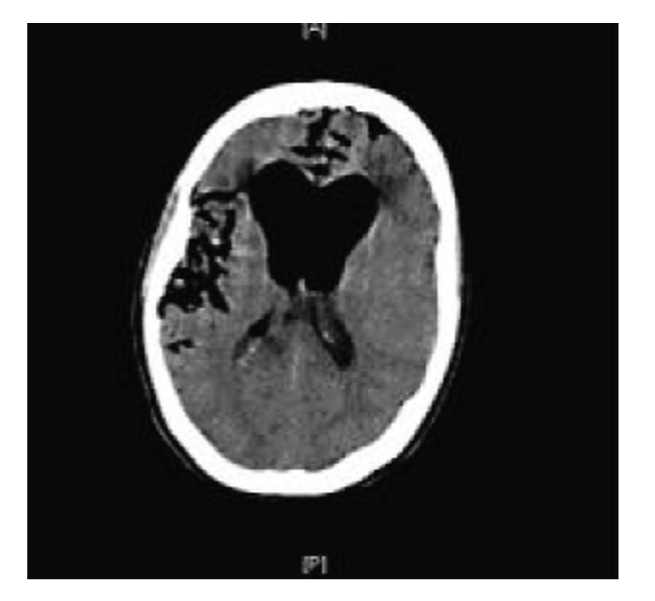
Noncontrast CT head scan showing extensive intraventricular and subarachnoid air.

Both attempts were unsuccessful and lumbar CSF drainage resulted in profound pneumocephalus (Figure 4). The patient suffered a rapid deterioration in clinical status, and following two generalised tonic-clonic seizures required intubation and ventilation in the neurointensive care unit. 

**Figure 5:**
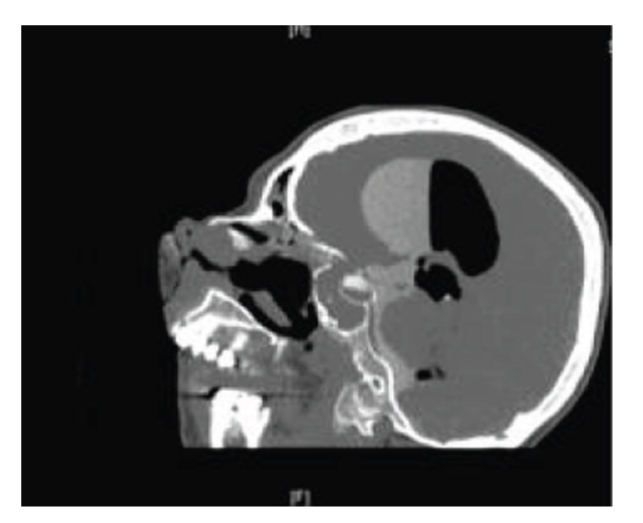
Sagittal reconstruction cisternogram image showing contrast in the sphenoid sinus consistent with persistent CSF leak.

Once sufficient neurological recovery had occurred, a second CT cisternogram (Figure 5) was undertaken prior to a further endoscopic endonasal repair using fat graft and fascia lata. In this procedure, 0 and 30° endoscopes (Karl Storz, Germany) and the Stealth Station Tria neuro-navigation system (Medtronic, USA) were used to locate and visualise the bony defects through a right-sided sphenoidotomy. Despite postoperative lumbar CSF drainage, this procedure was also unsuccessful.

### 2.2. Transcranial Repair

Following the fourth unsuccessful attempt, the patient underwent a transcranial repair of the CSF leak through a right-sided pterional craniotomy. Intraoperatively, no dural defect was visible; however, bony defects in the anterior pituitary fossa floor were palpable and therefore sealed with layers of temporalis fascia and muscle grafts secured with Tisseel sealant. A prolonged post-operative period of lumbar CSF drainage (7 days) was also undertaken which resulted in a successful control of CSF rhinorrhoea. 

## 3. Discussion

### 3.1. Histological Analysis—Olfactory Neuroblastoma versus Prolactinoma

**Figure 6:**
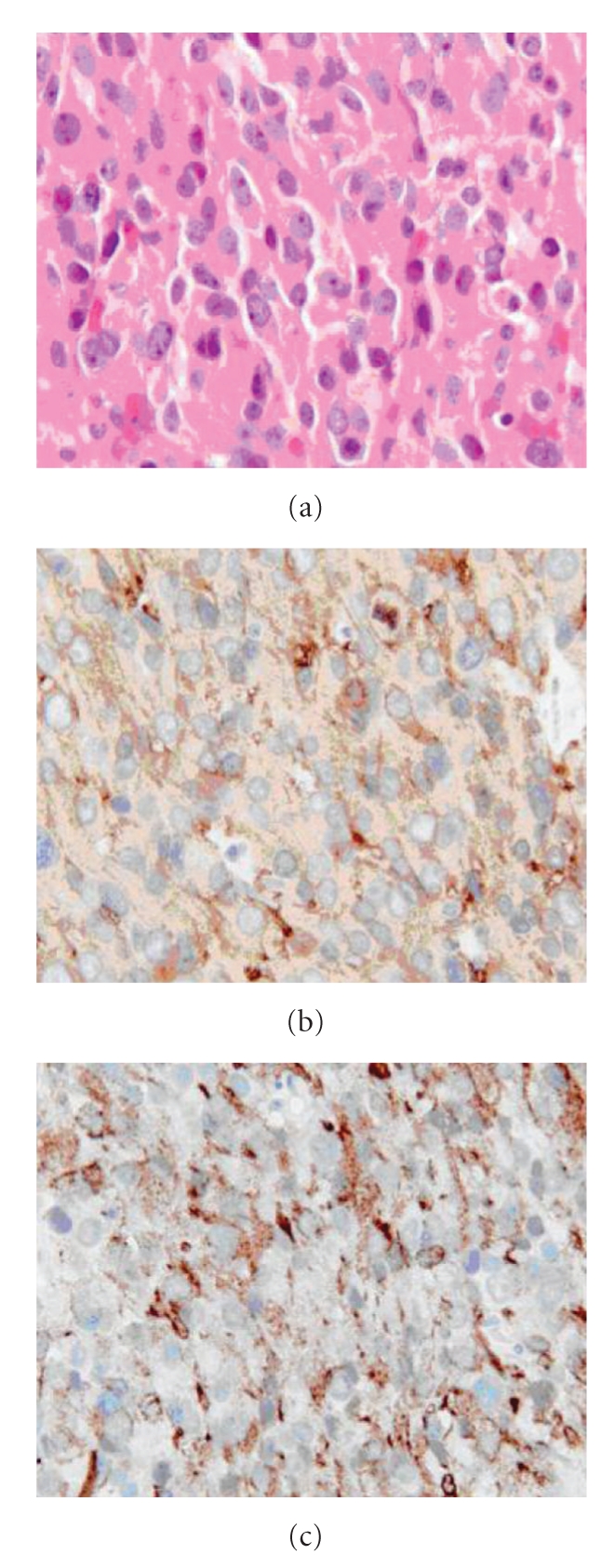
Histopathological section from the endoscopic biopsy showing sheets of cells with uniform round to oval nuclei and abundant eosinophilic cytoplasm (a), with widespread synaptophysin (b), and prolactin (c) immunoreactivity. Magnification x400.

The initial and subsequent biopsies were reviewed and showed an identical tumour consisting of sheets of cells with mildly pleomorphic round to oval nuclei and moderate amounts of eosinophilic cytoplasm (Figure 6(a)). Mitotic figures, tumour necrosis, and neuroblastic rosettes were not seen. Following immunocytochemistry, the tumour showed diffuse granular cytoplasmic immunoreactivity with antibodies to synaptophysin (Figure 6(b)) and prolactin (Figure 6(c)). Antibodies to the remaining anterior pituitary hormones and melanoma markers (mel-A, HMB-45 and S100) were all negative. The morphological findings and prolactin-immunoreactivity indicate a prolactinoma.

The difficulties encountered in definitive occlusion of the bony defects resulting in CSF rhinorrhoea in this case are likely to have resulted from a number of causes. Whilst bony destruction is a recognised feature of aggressive prolactinomas, the extensive infrasellar growth pattern seen in this case is unusual [1]. Once the diagnosis of prolactinoma was made, the patient was commenced on cabergoline, which in turn may have exacerbated the rhinorrhoea by reducing tumour bulk and revealing further bony defects. Finally, recurrent meningitis can result in impediment of normal CSF absorption pathways [2], which could have caused elevated CSF pressures and continued rhinorrhoea. Certainly CSF pressure on insertion of lumbar drains was always noted to be high, in spite of continued rhinorrhoea.

The potential pitfalls in differentiating olfactory neuroblastoma from prolactinoma and other paranasal tumours on histological examination have previously been described [3]. The histological diagnosis can be difficult, especially with relatively small biopsies, and in this case the presence of synaptophysin immunoreactivity led to the misdiagnosis of an olfactory neuroblastoma. However, pituitary adenomas usually also show immunoreactivity with neuronal markers [4], and the correct diagnosis required prolactin immunocytochemistry which was not available at the original histopathology department. Such cases should be referred for review by a specialist neuropathologist.

Complex paranasal and skull base lesions can result in bony defects and problematic CSF leaks. Although dopamine receptor agonists are the treatment of choice for prolactinomas, tumour shrinkage can take several weeks and residual tumour may contribute to recurrence of CSF leakage. Consequently, tumour debulking might be considered in patients with recurrent CSF rhinorrhoea in whom a histological diagnosis of prolactinoma has been confirmed. 

This case illustrates some of the potentially devastating complications of untreated CSF rhinorrhoea including recurrent meningitis and consequent raised CSF pressure, and pneumocephalus resulting in seizures and reduced conscious level. Management of these lesions requires a multidisciplinary approach involving ENT surgeons, neurosurgeons, neuroradiologists, and endocrinologists. The time at which endoscopic or transphenoidal approaches are abandoned in favour of transcranial surgery will depend on the surgical experience of the team and the anatomy of the site of CSF leakage. Despite advances in neuro-navigation and endoscopic surgery, transcranial repair maybe the only successful solution.
